# Comparative Analysis of Cardiovascular Development Related Genes in Stem Cells Isolated from Deciduous Pulp and Adipose Tissue

**DOI:** 10.1155/2014/186508

**Published:** 2014-12-07

**Authors:** Zhang Xin Loo, Wijenthiran Kunasekaran, Vijayendran Govindasamy, Sabri Musa, Noor Hayaty Abu Kasim

**Affiliations:** ^1^Department of Paediatric Dentistry and Orthodontics, Faculty of Dentistry, University of Malaya, 50603 Kuala Lumpur, Malaysia; ^2^Hygieia Innovation Sdn. Bhd (852106-M), Lot 1G-2G, Lanai Complex No. 2, Persiaran Seri Perdana, Precint 10, 62250 Putrajaya, Malaysia; ^3^Departments of Restorative Dentistry, Faculty of Dentistry, University of Malaya, 50603 Kuala Lumpur, Malaysia

## Abstract

Human exfoliated deciduous teeth (SHED) and adipose stem cells (ASC) were suggested as alternative cell choice for cardiac regeneration. However, the true functionability of these cells toward cardiac regeneration is yet to be discovered. Hence, this study was carried out to investigate the innate biological properties of these cell sources toward cardiac regeneration. Both cells exhibited indistinguishable MSCs characteristics. Human stem cell transcription factor arrays were used to screen expression levels in SHED and ASC. Upregulated expression of transcription factor (TF) genes was detected in both sources. An almost equal percentage of > 2-fold changes were observed. These TF genes fall under several cardiovascular categories with higher expressions which were observed in growth and development of blood vessel, angiogenesis, and vasculogenesis categories. Further induction into cardiomyocyte revealed ASC to express more significantly cardiomyocyte specific markers compared to SHED during the differentiation course evidenced by morphology and gene expression profile. Despite this, spontaneous cellular beating was not detected in both cell lines. Taken together, our data suggest that despite being defined as MSCs, both ASC and SHED behave differently when they were cultured in a same cardiomyocytes culture condition. Hence, vigorous characterization is needed before introducing any cell for treating targeted diseases.

## 1. Introduction

Heart disease is the leading cause of morbidity and mortality worldwide. The loss of cardiomyocytes and insufficient as well as delayed generation of cardiomyocytes upon onset of myocardial infarction rapidly result in the loss of heart function. Heart transplant and surgical intervention can prolong the life of a patient, but they do not address the fundamental issue, which is the replacement or regeneration of cardiomyocytes [1–3]. As a result, stem cell therapy has emerged as an alternative option with potential benefits for patients with end-stage heart disease. Embryonic stem cells (ESCs) have the capacity to generate any type of cell in the body due to its pluripotency in nature [4]. A previous study shows that ESCs were able to generate cardiomyocytes and had limited death rate in a rat heart ischemia model [5]. Nevertheless, a battery of pitfalls restricts the usage of this cell line in therapeutic application, namely, ethical issues involving destruction of embryo, complicated isolation methods, and the tendency to form tumours [6]. Cardiac stem cells (CSCs) offer better prospects and currently five different types of CSCs, including the c-kit^+^/Lin^−^ cells; the Sca-1^+^ cells; the Isl 1^+^ cells; the cardiac side population (Abcg2^+^/MDR^+^); and cardiosphere-derived stem cells (c-kit^+^/Sca-1^+^/Flk1^+^), have been identified [7–9]. Furthermore, a successful clinical trial using CSCs in human subjects with ischemic cardiomyopathy had been reported [10]. However, invasive procedures in isolating and culturing the cells coupled with escalating production cost due to autologous settings may hamper the reproducibility of such a trial in the future. This opens up an avenue for the usage of adult stem cells in treating cardiovascular diseases. Bone marrow derived mesenchymal stem cells (BM-MSCs) are the forerunning candidate in cardiac treatment (http://www.clinicaltrials.gov/). Interestingly, recent studies have shown that human dental pulp stem cells (DPSC) and human adipose stem cells (ASC) have been proven to generate cardiac-like cells which are able to improve heart function when delivered to rat* in vivo* [11, 12]. DPSC originate from the neural crest and ASC from the perivascular niche [13, 14]. This indicates that adult stem cells inherently carry genes that are related to cardiac cells although they originate from different parts of the body. In cardiogenesis, apart from the involvement of cardiac related genes, several transcription factors are reported to be involved as well. Transcription factors are DNA binding proteins that regulate gene expression by cooperating with the RNA polymerase II enzyme to synthesize messenger RNA molecules which are then used to produce proteins [15]. Members of cardiac related transcription factors include the Mef2 family, GATA family, Nkx-2 family, and Tbx family [16]. A past study has shown that overexpression of TBX5, GATA4, and MEF2C transcription factors in cardiac fibroblasts was able to generate cardiomyocytes [17]. This indicates the importance of transcription factors in determining cell fate. Nevertheless, to the best of our knowledge, there is no existing information on the basal expression of cardiac transcription factors in extracted deciduous pulp (SHED) and ASC. Hence, this experiment was carried out to investigate the transcription factors expressed in the cardiovascular development pathway. Further, based on the analysis of transcription factors, we stimulated the cells to undergo cardiac differentiation. This information will contribute more to our current understanding of the molecular events that takes place in SHED and ASC.

## 2. Materials and Methods

### 2.1. Tissue Collection and Isolation of Cells

This study was approved by the Medical Ethics Committee, Faculty of Dentistry, University of Malaya Medical Ethics Clearance number: DFCD0907/0042[L], and all donors provided written consent. Dental pulp stem cells were obtained from deciduous teeth (SHED; *n* = 3; age 5-6 years) and adipose stem cells (ASC) were extracted from subcutaneous adipose tissue of healthy donors undergoing fat removal for aesthetic purposes (*n* = 3; age 25–35 years). SHED and ASC were established as previously described [18, 19]. All cells were cultured using identical culture conditions: in T75 cm^2^ culture flasks (BD Pharmingen, San Diego, CA, USA) with culture medium containing 0.5% KO-DMEM (Invitrogen, Carlsbad, CA) and 200 units/mL and 200 *μ*g/mL penicillin/streptomycin (Invitrogen), 1% 1X Glutamax (Invitrogen), and 10% FBS (Thermo Fisher Scientific Inc.); humidified atmosphere of 95% air and 5% CO_2_ at 37°C; and cell seeding density of 1000 cells/cm^2^. Nonadherent cells were removed 48 hours after the initial plating. The medium was replaced every 3 days until the cells reached 80–90% confluence.

### 2.2. Multilineage Differentiation Assay

Adipogenic, chondrogenic, and osteogenic differentiation of SHED and ASC were carried out as previously described at the third passage [19]. The cultures were initiated at a density of 1000 cells/cm^2^ in six-well plates and grown until confluence and then subjected to differentiation into adipogenic, chondrogenic, and osteogenic lineages. Briefly, the adipogenic lineage was initiated by inducing the cells with 10% FBS, 200 *μ*M indomethacin, 0.5 mM 3-isobutyl-1-methylxanthine (IMBX), 10 *μ*g/mL insulin, and 1 *μ*M dexamethasone (all from Sigma-Aldrich). Lipid droplets were visualized using oil red staining (Sigma-Aldrich). For chondrogenic differentiation, cells were cultured in medium supplemented with ITS+1 (Sigma-Aldrich), 50 *μ*M L-ascorbic acid 2-phosphate, 55 *μ*M sodium pyruvate (Invitrogen), 25 *μ*M L-proline (Sigma-Aldrich), and 10 ng/mL transformation growth factor-*β* (TGF-*β*; Sigma-Aldrich). Assessment of proteoglycan accumulation was visualized by Alcian Blue staining (Sigma-Aldrich). Osteogenic differentiation was stimulated in a 3-week culture period in medium supplemented with 10% FBS, 10–7 M dexamethasone, 10 mM glycerol phosphate (Fluka, Buchs, Switzerland), and 100 *μ*M L-ascorbic acid 2-phosphate. The assessment of calcium accumulation was visualized using von Kossa staining (Sigma-Aldrich).

### 2.3. Flow Cytometry Analysis

Fluorescence activated cell sorting (FACS) was carried out as described previously in our paper [20]. The following antibodies were used to mark the cell surface epitopes: CD90-phycoerythrin (PE), CD44-PE, CD73-PE, CD166-PE and CD34-PE, CD45-fluoroisothyocyanate (FITC), and HLA-DR-FITC (all from BD Pharmingen). All analyses were standardized against negative control cells incubated with isotype specific IgG1-PE and IgG1-FITC (BD Pharmingen). At least 10,000 events were acquired on Guava Technologies flow cytometer, and the results were analyzed using Cytosoft, Version 5.2 (Guava Technologies).

### 2.4. Human Stem Cell Transcription Factor Array

RNA was extracted using RNeasy Mini Kit (Qiagen) and was then treated with DNase to remove genomic DNA before quantified using NanoDrop 2000 (Thermo Fisher Scientific Inc.). After that, extracted RNA was reverse-transcribed into cDNA using RT2 First Strand Kit (Qiagen) according to the manufacturer's instructions. cDNA was then loaded onto the array for thermal cycling on an ABI PRISM 7500 Fast Sequence Detection System (Applied Biosystems). A cutoff cycle threshold (Ct) value of 39 was arbitrarily assigned where a Ct value above 39 was considered to be undetected. The levels of gene expression for SHED (target) relative to the level of expression in ASC (calibrator) and vice versa were analyzed using comparative Ct method (ΔΔCt).

### 2.5. Validation of Human Stem Cell Transcription Factor Array Gene Expression by Reverse Transcriptase and Real-Time PCR

cDNA amplification was performed in a thermocycler using Taq polymerase supplied with KCl buffer and 1.5 mM/L MgCl_2_ (Invitrogen) at 94°C/1 min, 58°C/30 sec, and 72°C/1 min. Polymerase chain reaction (PCR) products were resolved onto 1.5% agarose (Invitrogen) gel in 1X Tris borate-ethylenediaminetetraacetic acid buffer. Primer sequences are shown in Table 1. The expressions of some of the primers in the reverse transcriptase PCR analysis were quantified in duplicate with SYBR green master mix (Applied Biosystems, Foster City, CA, USA). PCR reactions were ran on an ABI 7500 Fast Sequence Detection System (Applied Biosystems), and all measurements were normalized by 18s rRNA. For data analysis, the comparative Ct method (ΔΔCt) was used.

### 2.6. Ingenuity Pathway Analysis

The “Core Analysis” function included in IPA (Ingenuity Systems Inc., California, USA; http://www.ingenuity.com/) was used to interpret the data in the context of biological processes, pathways, and networks. Upregulated genes with at least a 2-fold change were selected for analysis. After the analysis, generated networks were ordered by score significance. Meanwhile, significance of the biological function was tested using *P* value from the right-tailed Fisher exact test. Physiological system development and function was chosen for analysis. Selected networks were then converted to form pathways via path designer to show relationships between genes and protein.

### 2.7. Directional Differentiation towards Cardiac-Like Cells

In brief, SHED and ASC were seeded onto 6-well plates (BD Biosciences) at a density of 100 000 cells/cm^2^. To induce cardiac differentiation, KO-DMEM (Invitrogen) containing 200 units/mL and 200 *μ*g/mL penicillin/streptomycin (Invitrogen) and 1% 1X Glutamax (Invitrogen) were supplemented with 100 ng/mL human recombinant activin A (R&D Systems) for 24 hours followed by 10 ng/mL human recombinant BMP 2 (R&D Systems) for 4 days. The medium was then exchanged for KO-DMEM without supplementary cytokines, and cultures were refed every 2-3 days in a humidified incubator at 37°C and 5% CO_2_ for up to 14 days. Cell morphology was captured using an inverted microscope.

### 2.8. Analysis of Cardiac Differentiation Using Cardiomyocyte Differentiation Arrays

Total RNA was isolated using RNeasy Mini Kit at day 7 and day 14. Extracted RNA was then treated with DNase to remove genomic residues and 1 *μ*g RNA was reverse-transcribed using RT2 First Strand Kit. cDNA was mixed with SYBR Green Rox Master Mix and loaded into cardiomyocyte differentiation arrays (all from Qiagen) for analysis of 19 cardiomyocyte specific markers using ViiA7 Fast Sequence Detection System (Applied Biosystems). Expression levels were calculated using the ΔΔCt method. A cutoff cycle threshold (Ct) value of 35 was arbitrarily assigned, such that a Ct value above 35 was considered to be undetected. GAPDH was used as a housekeeping gene for data normalization purposes.

### 2.9. Immunocytochemistry

Differentiated cells were fixed with 4% paraformaldehyde for 15 minutes and permeated using 0.1% Triton X-100 for 10 minutes, both at room temperature. Cells were then blocked with blocking buffer (DPBS containing 3% BSA) for 30 minutes at room temperature and incubated overnight with primary antibodies at 4°C. Primary antibodies used were rabbit polyclonal to GATA4, mouse monoclonal to NKX2.5, and mouse monoclonal to *α*-actinin (Abcam). After three washes with DPBS, cells were incubated with secondary antibodies Alex flour 594-conjugated anti-rabbit IgG antibodies (Molecular probes) for GATA4 and FITC-conjugated anti-mouse IgG antibodies (Abcam) for NKX2.5 and *α*-actinin. Nuclei were stained with 4′,6′-diamidino-2-phenylindole dihydrochloride (DAPI; Chemicon) at a dilution of 1 : 500 for 30 minutes at room temperature. Observations were then made using a fluorescent microscope.

### 2.10. Statistical Analysis

Data are presented as mean + standard deviation (SD). Statistical comparisons were made using Student's *t*-test and values of *P* < 0.05 were considered significant.

## 3. Results

### 3.1. Characterization of MSCs Derived from Deciduous Pulp and Adipose Tissue

**Figure 1:**
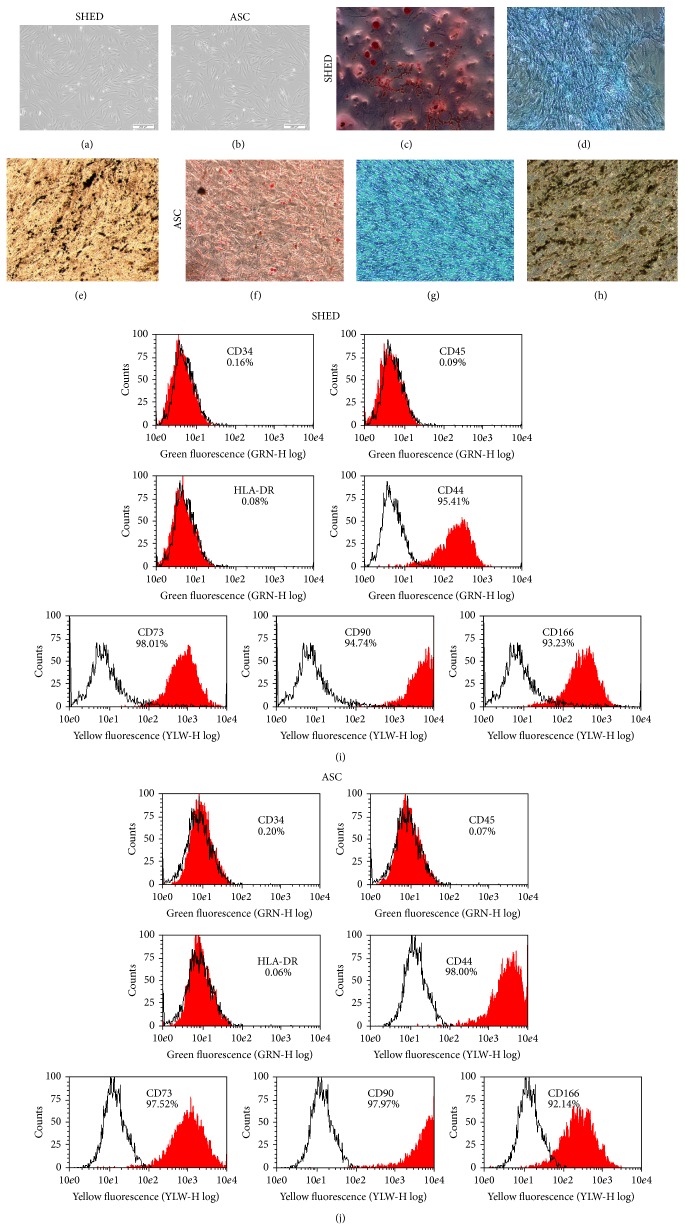
(a-b) Morphology of SHED and ASC using phase contrast microscope at 10x magnification.* In vitro *mesoderm differential potential of SHED and ASC. Adipogenesis was detected by neutral oil droplet formation stained with oil red O. Chondrogenesis was detected by the presence of proteoglycans stained with Alcian Blue. Osteogenesis was confirmed by mineralized matrix deposition stained with von Kossa staining. All staining processes were done 21 days after induction for SHED (c–e) and ASC (f–h). (i-j) Immunophenotype analysis of SHED and ASC. Cells were tested against human antigens CD34, CD44, CD45, CD73, CD90, CD166, and HLA-DR. All experiments were conducted at passage 3 with 3 biological replicates for each established cell line. The percentages of positive cells shown in the figure are average of the three donors.

In order to ascertain that the cell lines that we established are bone fide MSCs, we performed some basic MSCs characterization studies. Both types of cells displayed fibroblastic morphology (Figures 1(a) and 1(b)). Next, we investigated the mesoderm differential potential of ASC and SHED into adipogenic, chondrogenic, and osteogenic lineages under appropriate media induction and they were able to undergo adipogenesis, chondrogenesis, and osteogenesis, respectively (Figures 1(c)–1(h)). To further characterize these cells, immunophenotyping was done by flow cytometry. Both samples were negative for hematopoietic markers CD34 and CD45, whereas more than 85% of the results were positive for MSC markers CD44, CD73, CD90, and CD166 (Figures 1(i) and 1(j)).

### 3.2. Snapshot of Potential Role of Transcription Factor

**Figure 2:**
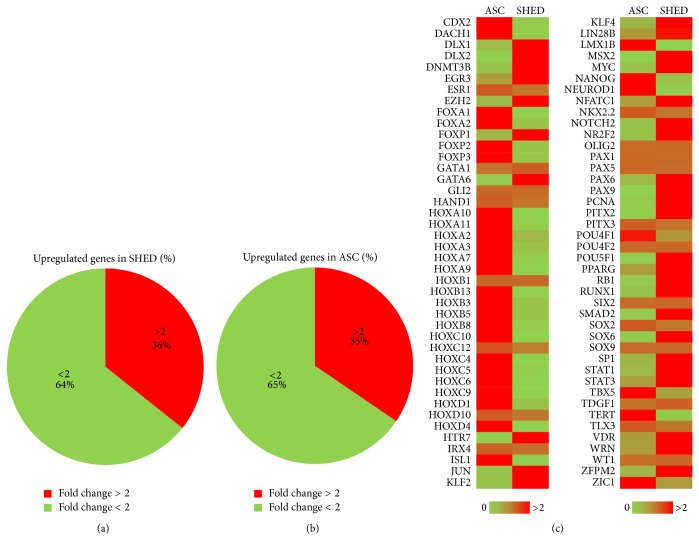
Comparison of gene profile between SHED and ASC. (a-b) Percentage of upregulated genes in ASC and SHED. (c) Genes are represented in the heat map; low expression in green and high expression in red.

We further characterized ASC and SHED using human stem cell transcription factor array before inducing cardiac differentiation. An almost equal percentage of upregulated transcription factor genes were detected in both sources. Percentages of gene expression levels which showed a more than 2-fold change were 35% for ASC and 36% for SHED (Figures 2(a) and 2(b)). Expressions of HOXA, HOXB, and HOXC group of genes were higher in the ASC. Meanwhile, SHED expressed many genes that are related to pluripotency such as DNMT3B, KLF4, MYC, and POU5F1 (Figure 2(c)).

### 3.3. Ingenuity Pathway Analysis

**Figure 3:**
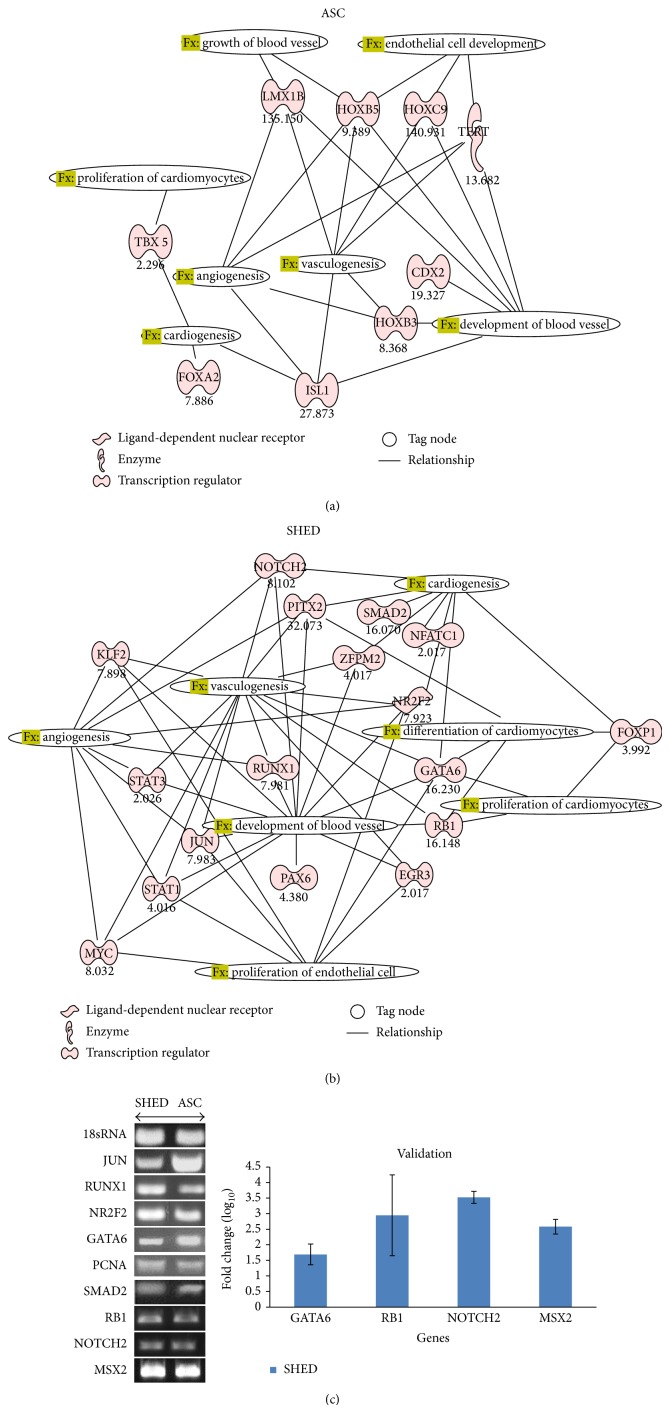
Transcription factors that are involved in cardiovascular-related development pathways in ASC and SHED. (a) Major pathways in ASC are development of blood vessel, vasculogenesis, angiogenesis, growth of blood vessel, cardiogenesis, endothelial cell development, and proliferation of cardiomyocytes. (b) Major pathways in SHED are development of blood vessel, vasculogenesis, cardiogenesis, angiogenesis, proliferation of cardiomyocytes and endothelial cells, and differentiation of cardiomyocytes. (c) Validation of gene expression by reverse transcriptase and real-time PCR. Reverse transcriptase of selected genes. mRNA expression of GATA6, RB1, NOTCH2, and MSX2 by real-time PCR using SYBR green reagent with values normalized to 18sRNA.

Information from pathway enrichment analysis showed that the transcription factor genes fall under several cardiovascular-related development processes, namely, growth and development of blood vessel, angiogenesis, vasculogenesis, proliferation, and differentiation of cardiomyocytes (Figures 3(a) and 3(b)). The main physiological system development and function pathways in SHED include organismal survival (average *P* = 1.94*E* − 5), digestive system development and function (8.90*E* − 5), cardiovascular system development and function (9.35*E* − 5), tissue morphology (8.10*E* − 5), embryonic development (1.29*E* − 5), and cellular moment (1.23*E* − 5), whereas, for ASC, the pathways are embryonic development (5.05*E* − 3), organismal development (4.40*E* − 3), skeleton muscle system development and function (4.35*E* − 3), organ morphology (5.20*E* − 3), tissue morphology (4.35*E* − 3), and cardiovascular system development and function (5.05*E* − 3) (Table 2). Some of the genes were selected randomly to validate gene expression of human stem cell transcription factor array by using reverse transcriptase and real-time PCR (Figure 3(c)).

### 3.4. Cardiovascular System Development and Function

In the context of cardiovascular system development and function, both types of stem cells displayed different functions. A total of 5 major pathways were chosen based on significance value. For SHED, the major pathways are development of blood vessel (1.64*E* − 13), vasculogenesis (1.12*E* − 12), cardiogenesis (1.73*E* − 9), angiogenesis (1.07*E* − 7), proliferation of cardiomyocytes and endothelial cells (1.29*E* − 7), and differentiation of cardiomyocytes (2.93*E* − 7). As for ASC, they are development of blood vessel (2.21*E* − 6), vasculogenesis (1.91*E* − 5), angiogenesis (1.78*E* − 4), growth of blood vessel (3.39*E* − 4), cardiogenesis (1.56*E* − 3), endothelial cell development (3.13*E* − 3), and proliferation of cardiomyocytes (4.64*E* − 2) (Table 3). To validate the result of the human stem cell transcription factor array analysis, GATA6, RB1, NOTCH 2, and MSX2 were randomly chosen for real-time PCR.

### 3.5. Cardiac Differentiations of ASC and SHED

**Figure 4:**
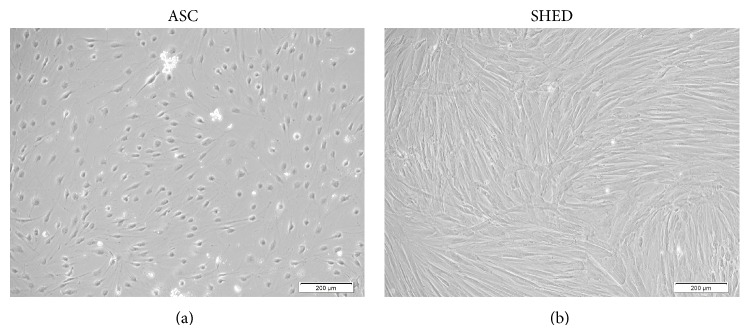
Differentiation of ASC and SHED into cardiomyocyte-like cells. (a-b) Images of ASC and SHED captured using phase contrast microscope at 10x magnification showing cell morphology after differentiation into cardiomyocyte-like cells.

**Figure 5:**
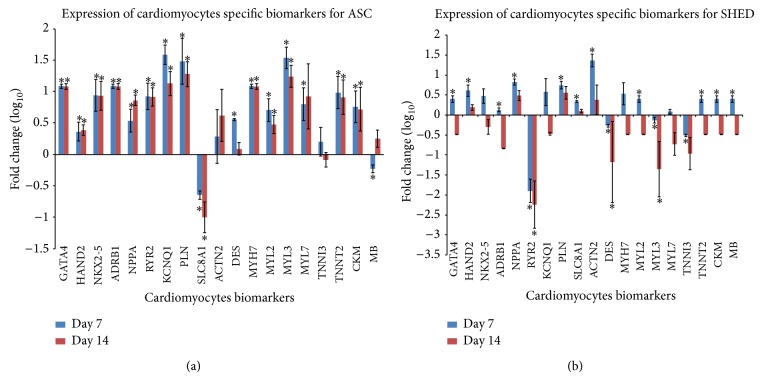
Expression of cardiomyocyte specific biomarkers. (a) Expression of cardiomyocyte specific biomarkers for ASC and (b) SHED undergoing cardiac differentiation at day 7 and day 14. GATA4, HAND2, and NKX2.5 are cardiomyocyte transcription factors; ADRB1, NPPA, and RYR2 are cardiomyocyte receptors; KCNQ1, PLN, and SLC8A1 are cardiomyocyte ion channels; ACTN2, DES, MYH7, MYL2, MYL3, and MYL7 are cardiomyocyte structural constituents; CKM and MB are cardiomyocyte enzyme and transporter. ^*^ *P* < 0.05.

**Figure 6:**
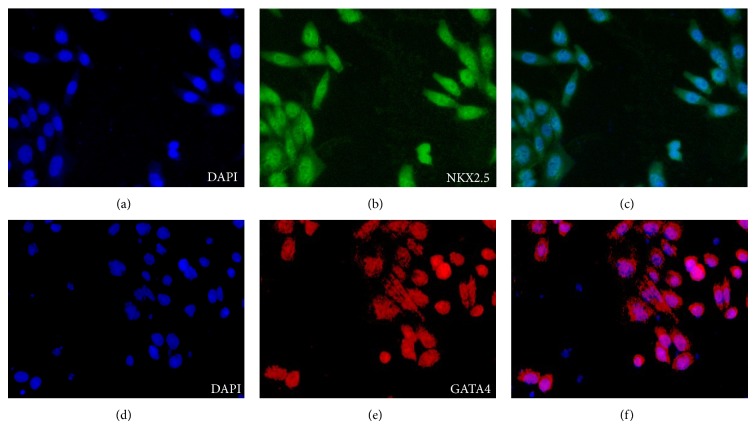
Immunocytochemistry of targeted transcription factors NKX2.5 and GATA4 in ACS. This was not detected in SHED. Expression of *α*-actinin was not detected in both samples.

Both ASC and SHED displayed different morphologies after 14 days of cardiac differentiation; ASC appeared to be polygonal, whereas SHED resembles a striated shape. Unfortunately, no spontaneous cellular beating was detected in both cell lines (Figure 4). A peeped into the gene expression profiling revealed that, in ASC cardiac induction, cardiomyocyte transcription factors, receptors, and ion channels were significantly upregulated after 14 days. Only SLC8A1 was markedly downregulated. In terms of cardiomyocyte structural constituent, MYH7, MYL2, and MYL3 were upregulated significantly (*P* < 0.05) after 14 days as opposed to DES and MYL7 which were only upregulated significantly (*P* < 0.05) at the 7th day (Figure 5(a)). As for SHED cardiac induction, only a handful of genes, namely, GATA4, HAND2, ADRB1, NPPA, PLN, SLC8A1, ACTN2, MLY2, TNNT2, MB, and CKM, were upregulated significantly (*P* < 0.05) at the 7th day. RYR2, DES, and MYL3 were significantly downregulated (*P* < 0.05) after 14 days (Figure 5(b)). These findings were further confirmed at protein level (Figure 6).

## 4. Discussion

Bone marrow derived stem cells (BM-MSCs) were regarded as the best adult cell source in treating cardiac related diseases, with an example given by a systematic review paper [21], whereby the results showed a moderate yet significant improvement in global heart function using BM-MSCs. Alternatively, dental and adipose derived stem cells have the potential to become prime candidates because they are biological waste products, have great capacity for proliferation, and do not require invasive procedures in obtaining MSCs. Therefore, we have undertaken the present work to study two different sources of MSCs from adipose tissue and dental pulp for the purpose of cardiac differentiation. MSCs derived from extracted deciduous teeth and adipose tissue that we obtained from our study conform to the proposed criteria by the Mesenchymal and Tissue Stem Cell Committee of the International Society for Cellular Therapy [22, 23]. The cells are fibroblastic; they express MSCs markers and are capable of differentiating into osteoblasts, adipocytes, and chondroblasts when cultured in inductive media.

Human stem cell transcription factor arrays were used to further characterize SHED and ASC. This is because a large amount of experimental work emphasized understanding stem cell characteristics such as proliferation pathways [24], quality of the cells under* in vitro* culture conditions [25], and expression pattern of Oct-4, Sox2, and c-Myc in primary culture of cells [26]. Proliferation rate should be taken into consideration because approximately one billion cells are needed to replace injured or dead cells at the infarct zone. There is a lack of information pertaining to stem cells at the basal level. Through this study, we found that there is a higher expression level for the HOX group of genes in the ASC. This could be due to the aforementioned group of genes which play a role in development of obesity and body fat distribution [27]. We found that SHED express many markers related to pluripotency and this result was in agreement with many other independent studies that have reported SHED to be more primitive in nature [28].

Further, based on the pathway analysis using Ingenuity programme, both types of cells are prone to angiogenesis as there are more interconnected molecules involved in formation of blood vessels and vasculogenesis. This observation was similar to the previous studies [11, 12]. This provides an explanation of how stem cells are able to improve or preserve cardiac function in an animal model through angiogenesis and not due to cardiomyocyte differentiation. The actual mechanism behind this is still uncertain. However, we postulate that hypoxic conditions play an important role. In hypoxic conditions, expression of hypoxia inducible factor-alpha increases rapidly which in turn activates genes that encode for proangiogenic growth factors, such as vascular endothelial growth factor, basic fibroblast growth factor, angiopoietin 1 and angiopoietin 2, placental growth factor, and platelet-derived growth factor-B [29]. These released cytokines encourage angiogenesis and the angiogenic effects of human multipotent stromal cell which explains the underlying improvement of cardiac function through any individual or a combination of mechanisms such as apoptosis inhibition, increase in survival, and angiogenesis stimulation by activation of the PI3K-Akt pathway [11, 30].

We then proceeded with inducing SHED and ASC into cardiomyocytes, as proliferation and differentiation involved in cardiomyocyte pathways were observed in the Ingenuity pathway analysis. Based on gene expression levels, ASC expressed significantly more cardiomyocyte specific biomarkers compared to SHED at both day 7 and day 14 of induction period. Higher expression of GATA4, HAND2, and NKX2.5 in ASC compared to SHED may be the underlying reason which leads to more stable gene expression that are related to structural constituents. Ieda et al. have shown that overexpression of GATA4, MEF2C, and TBX5 in cardiac fibroblasts led to cardiomyocyte generation. The GATA proteins are a family of zinc finger-containing transcription factors. Three GATA family members, GATA4, GATA5, and GATA6, have been detected in presumptive heart cells and have been shown to activate numerous myocardial genes. GATA4 and GATA6 are both transcription factors in the GATA family and play a role in cardiomyocyte development [16]. Absence of both GATA4 and GATA6 blocks cardiac myocyte differentiation and results in acardia in mice, and a particular threshold for GATA4 and GATA6 expression is required for cardiovascular development [31, 32]. This clearly indicates that not all sources of MSCs are suitable for cardiac differentiation and the selection of the MSC source for treating a disease highly depended on its origin. Hence, in our study, we found that ASC express many cardiomyocytes related genes simply because both adipose and heart development are mesoderm origin.

Spontaneous cellular beating, which is the hallmark indicator of successful cardiomyocyte differentiation, was unfortunately not detected in our experiment. This could be explained by the fact that cardiomyogenic marker troponin I was suppressed in this condition whereby it can only be activated when the cells were cultured with neonatal rat cardiomyocytes, or when these cells were delivered* in vivo* to murine models of myocardial infarction [33]. In ASC differentiation, it assumed more of a polygonal shape which is similar to the morphology [34]. They managed to obtain beating cardiomyocytes by coculturing the cells with contracting cardiomyocytes as cell-to-cell interaction was identified as a key inducer for cardiomyogenic differentiation. Limitations of this protocol include the absence of an appropriate clue as to what triggers the cells are to differentiate, producing cardiac phenotype or beating, and whether rat cardiomyocytes were used. There have been no reports yet of a successful protocol using dental stem cells to generate beating cardiomyocytes. Prior examples utilized dental pulp stem cells with a combination of vascular endothelial growth factor, basic fibroblast growth factor, and insulin-like growth factor 1. In this case, spontaneous cellular beating was also not detected, and the morphology of the cells was reported to be elongated and irregular, similar to what we observed in our experiments [35]. In our cardiac differentiation protocol, we used a combination of activin A and BMP 2. The reason behind this is that stem cell differentiation in the heart requires a paracrine pathway and BMP 2 is one of the contributing factors [36] and fine tuning this differentiation method may lead to the improvement of cardiac maturation [37].

In conclusion, although we have identified a number of transcriptional factors related to cardiac development in ASC and SHED, ASC are a better choice of cells than SHED in treating cardiac related diseases. Our findings provide a basis as to the presence of variations in cardiomyocytes generated from MSC sources and contribute to a better understanding of the fundamental concepts in the application of stem cells for the treatment of cardiac diseases.

## Figures and Tables

**Table 1:** List of primers used in this study.

Gene symbol	Forward/	Base pairs
(gene bank)	reverse	
18sRNA	CGGCTACCATCCAAGGAA	186
	GCTGGAATTACCGCGGCT	

RUNX1 (NM_001754)	GTGGTCAGCAGGCAGGACGAA	678
	TGGCGACTTGCGGTGGGTTTG	

JUN (NM_002228)	CGCTGCCTCCAAGTGCCGAA	673
	GCCTCGCTCTCACAAACCTCCC	

NR2F2 (NM_021005)	CGAGTGCGTGGTGTGCGGAG	766
	GGCTACATCAGAGAGACCACAGGCA	

GATA6 (NM_005257)	CGGCGGCTTGGATTGTCCTGTG	663
	GCCCTTCCCTTCCATCTTCTCTCA	

PCNA (NM_182649)	GCAGGCGTAGCAGAGTGGTCG	482
	AACTTTCTCCTGGTTTGGTGCTTCA	

SMAD2 (NM_005901)	GTTCCGCCTCCAATCGCCCA	209
	TGGCGGCGTGAATGGCAAGA	

RB1 (NM_000321)	GCAACCTCAGCCTTCCAGACCC	705
	GCTTCCTTCAGCACTTCTTTTGAGC	

NOTCH2 (NM_024408)	TGGCTTTGCTGGGGAGCGTTG	654
	CAGGGAAGTGGGGAGGAGCAGT	

MSX2 (NM_002449)	AGATGGAGCGGCGTGGATGC	660
	GGCTTGGTGCCTCCGCCTAC	

**Table 2:** Top physiological system development and function in SHED and ASC.

Physiological system development and function					
SHED			ASC		
Function	Highest *P*value Lowest *P* value	Molecules	Function	Highest *P* value Lowest *P* value	Molecules
Organismal survival	1.40*E* − 17 3.88*E* − 05	27	Embryonic development	1.74*E* − 28 1.01*E* − 02	24

Digestive system development and function	1.19*E* − 15 1.78*E* − 04	18	Organismal development	1.74*E* − 28 8.80*E* − 03	25

Cardiovascular system development and function	4.77*E* − 15 1.87*E* − 04	22	Skeletal and muscular system development and function	2.95*E* − 19 8.70*E* − 03	19

Tissue morphology	4.73*E* − 13 1.62*E* − 04	23	Organ morphology	2.27*E* − 18 1.04*E* − 02	23

Embryonic development	5.72*E* − 13 2.57*E* − 04	28	Tissue morphology	7.73*E* − 18 8.70*E* − 03	25

Cellular movement	7.37*E* − 13 2.46*E* − 04	23	Cardiovascular system development and function	2.80*E* − 06 1.01*E* − 02	14

**Table 3:** Top functions in the cardiovascular system development and function in SHED and ASC.

Cardiovascular system development and function			
SHED (22)		ASC (14)	
Function	*P* value (molecules)	Function	*P* value (molecules)
Development of blood vessel [EGR3, GATA6, JUN, KLF2, MYC, NOTHC2, NR2F2, PAX6, PITX2, RB1, RUNX1, STAT1, STAT3, ZFPM2]	1.64*E* − 13 (14)	Development of blood vessel [CDX2, HOXB3, HOXB5, HOXC9, ISL1, TERT, LMX1B]	2.21*E* − 6 (7)

Vasculogenesis [EGR3, GATA6, JUN, KLF2, MYC, NOTCH2, NR2F2, PITX2, RB1, RUNX1, STAT1, STAT3, ZFPM2]	1.12*E* − 12 (13)	Vasculogenesis [HOXB3, HOXB5, HOCX9, ISL1, TERT, LMX1B]	1.91*E* − 5 (6)

Cardiogenesis [FOXP1, GATA6, NFATC1, NOTCH2, NR2F2, PITX2, SMAD2, ZFPM2]	1.73*E* − 9 (8)	Angiogenesis [HOXB3, HOXB5, ISL1, TERT, LMX1B]	1.78*E* − 4 (5)

Angiogenesis [JUN, KLF2, MYC, NOTHC2, NR2F2, PITX2, RUNX1, STAT1, STAT3]	1.07*E* − 7 (9)	Growth of blood vessel [HOXB5, LMX1B]	3.39*E* − 4 (2)

Proliferation of cardiomyocytes [FOXP1, GATA6, RB1] and endothelial cells [EGR3, GATA6, JUN, KLF2, MYC, NR2F2, STAT1]	1.29*E* − 7 (3) and (7)	Cardiogenesis [FOXA2, ISL1, TBX5]	1.56*E* − 3 (3)

Differentiation of cardiomyocytes [FOXP1, GATA6, PITX2, RB1]	2.93*E* − 7 (4)	Endothelial cell development [HOXB5, HOXC9, TERT]	3.13*E* − 3 (3)

—		Proliferation of cardiomyocytes [TBX5]	4.64*E* − 2 (1)
